# Numerical investigation on functional limitations of the anti-stall fin for an axial fan: one-factor analyses

**DOI:** 10.1038/s41598-022-19530-9

**Published:** 2022-09-09

**Authors:** Yong-In Kim, Hyeon-Mo Yang, Kyoung-Yong Lee, Young-Seok Choi

**Affiliations:** 1 Industrial Technology (Green Process and Energy System Engineering), University of Science and Technology, Daejeon, South Korea; 2 Carbon Neutral Technology R&D Department, Research Institute of Clean Manufacturing System, Korea Institute of Industrial Technology, Cheonan, South Korea

**Keywords:** Mechanical engineering, Fluid dynamics, Scientific data

## Abstract

The stall in an axial fan is directly related to detrimental phenomena such as performance degradation, vibration, noise, and flow instability at low flow rates. As a kind of passive control method to handle the stall, two-dimensional plates so-named anti-stall fin (ASF) were suggested by ourselves and were attached inside the casing. In this study, the ASF's effect on the internal flow pattern was visually investigated in the flow passage, and its tendency was discussed with the performance curve. Subsequently, the ASF's functional limitations for various design parameters, which the ASF can derive aerodynamically, were presented as the primary focus of this study. Each one-factor analysis was performed, and the internal flow pattern was observed in parallel at the point where the ASF lost its function. For the radial length, axial length, number of fins, and positive-tangential angle, the ASF almost retained its function up to the limitation to prevent instability but radically lost its function at a certain flow rate. For the axial gap and negative-tangential angle, the ASF gradually lost its function. Mostly, this study was based on numerical analysis, and the performance was validated through experimental tests.

## Introduction

In the lower flow rates of fluid machinery, ‘stall’ is one of the most detrimental phenomena that has various instabilities due to an increase in incidence angle. Based on the theoretical and empirical discussion as well known in our field, unfavorable factors that can be contained in the stalling flow rates are as follows: positive gradients (degradation) on performance curve ($Q$–$P$ or $\varPhi$–$\varPsi$)^1,2^; backflow and rotating stall inside inlet passage^3,4^; blade fluctuating stress^5^; pressure fluctuation^6^; vibration^7,8^; noise^9,10^. Here, the backflow should be developed from the blade (rotor) leading edge (LE) and gradually increases in the spanwise and streamwise direction as the flow rate decreases, whereas the intensity for the other factors such as pressure fluctuation, vibration, and noise may not be inversely proportional to the flow rate. Regardless of each intensity, if these factors in the stalling flow rates are suppressed without any instability, an efficient operation can be secured through the expansion of the stall margin. A stall-free system is available to adjust the flow rate wider.

Accordingly, researchers have been trying to control the stall for decades. Their deep efforts eventually paid off in the anti-stalling performance; however, each of them might face major or minor disadvantages in the case by case: operating devices and systems; cost and time; complicated design; installation space and maintenance; performance degradation (or change) from design specification. These disadvantages make each method for controlling stall hesitant to be actively applied in industrial fields. The stall needs to be controlled in a more practical and simple way.

As a kind of passive control method by ourselves, two-dimensional plates so-named anti-stall fin (ASF) were suggested to be attached inside the inlet casing and toward the shaft^11,12^. In the designing process, the ASF's axial directionality (angle; $\beta$) was not considered because it inevitably causes the absolute flow angle at the blade inlet and leads to the decrease (or change) in performance even near the design flow rate, i.e., the ASF exhibited two-dimensional geometry. The features that could be obtained with this method were as follows: no operating devices and systems; no additional space; simple configuration; immediacy (on-site welding or fastening; semi-permanent); guaranteed performance based on design specification; regardless of material (iron, rubber, plastic, etc.). Above all, this method thoroughly succeeded in suppressing the positive gradients on the $Q$–$P$ curve; that is, suppression of stall-induced instabilities was expected with the ASF. Here, it is required to consider the functional limitations of anti-stalling performance.

In this study, the effect of ASF on the internal flow pattern was investigated in the flow passage, and its tendency was discussed with the performance curve. Subsequently, the functional limitations of ASF were analyzed and suggested as the primary focus of this study. Here, a kind of one-factor analysis was performed for various design parameters that the ASF can derive aerodynamically, and the internal flow pattern was observed in parallel at the point where the ASF lost its function. The design parameters were selected as radial length, axial length, axial gap, number of fins, and positive- and negative-tangential angle of the ASF. The evaluation of ASF's functional limitation was based on the following declaration: ‘negative gradients in the flow rate range more than 0.5 ${\varPhi }_{d}$ on the $Q$–$P$ curve’. Additional discussion was accompanied on how the ASF tends to lose its function for each parameter. Mostly, this study was based on numerical analysis, and the performance before/after the application of ASF was validated through experimental tests. Since this study was on the low flow rates, which are generally difficult to numerically converge, a modified turbulence model was applied as a specified method in the numerical analysis. The results are expected to serve as basic data for the recently originated ASF. If there are restrictions on each variable in applying ASF, the results can be worth referring to our field.

**Figure 1:**
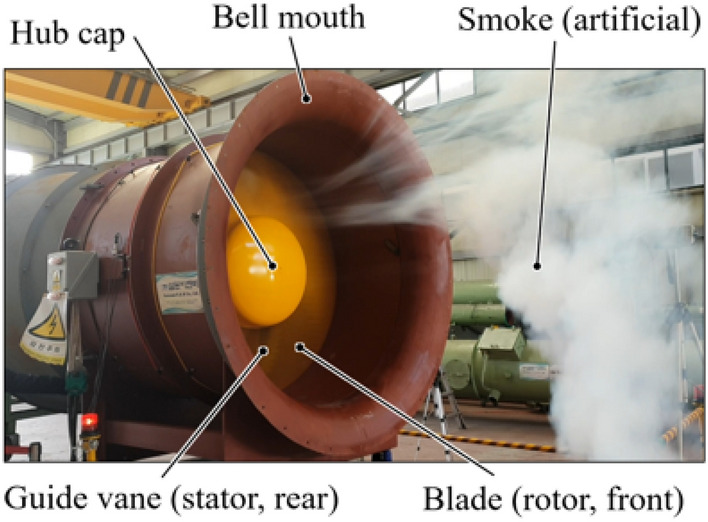
Typical assembly of an axial fan.

Meanwhile, the axial fan for applying the ASF was being used in the general industry, as shown in Fig. 1; it is a prototype of this study. Table 1 lists the design specifications and parameters, where $\omega$, $Q$, $P$, $\rho$, $c$, $u$, $r$, ${\delta }_{t}$, $D$, $C$, and $S$ denote the angular velocity, volume flow rate, total pressure, air density at 25 °C, absolute velocity, circumferential velocity, fan radius, tip clearance, fan diameter, blade chord length, and blade pitch, respectively, and subscripts 2, $m$, $h$, and $s$ denote blade outlet, meridional component, fan hub, and fan shroud, respectively; here, ${u}_{2}$ was assigned as the blade tip, and subscript d could imply the design point but omitted, e.g., ${\varPhi }_{d}=$ 0.285.

**Table 1:** Design specifications and parameters of the axial fan.

Specification or parameter	Value [unit]	Definition
Specific speed^a^ (${N}_{s}$)	7.77 [−]	$(\omega \sqrt{Q})/{(P/\rho )}^{0.75}$
Flow coefficient ($\varPhi$)	0.285 [−]	${c}_{m2}/{u}_{2}$
Pressure coefficient ($\varPsi$)	0.117 [−]	$P/(0.5\rho {{u}_{2}}^{2})$
Rotational speed ($N$)	1470 [revolutions-per-minute]	
Hub ratio	0.44 [−]	${r}_{h}/{r}_{s}$
Tip clearance^b^ ratio	0.01 or 0.0028 [−]	${\delta }_{t}/({r}_{s}-{r}_{h})$ or ${\delta }_{t}/{D}_{2}$
Solidity	0.769 (hub), 0.155 (shroud) [−]	$C/S$
Setting angle^c^	49.7 (hub), 23.1 (shroud) [°]	
No. of blades & guide vanes	10 & 11	
Airfoil series of blade	NACA 3512	

^a^Type number.

^b^Between a blade tip and casing.

^c^Tangential definition.

## Design parameters

**Figure 2:**
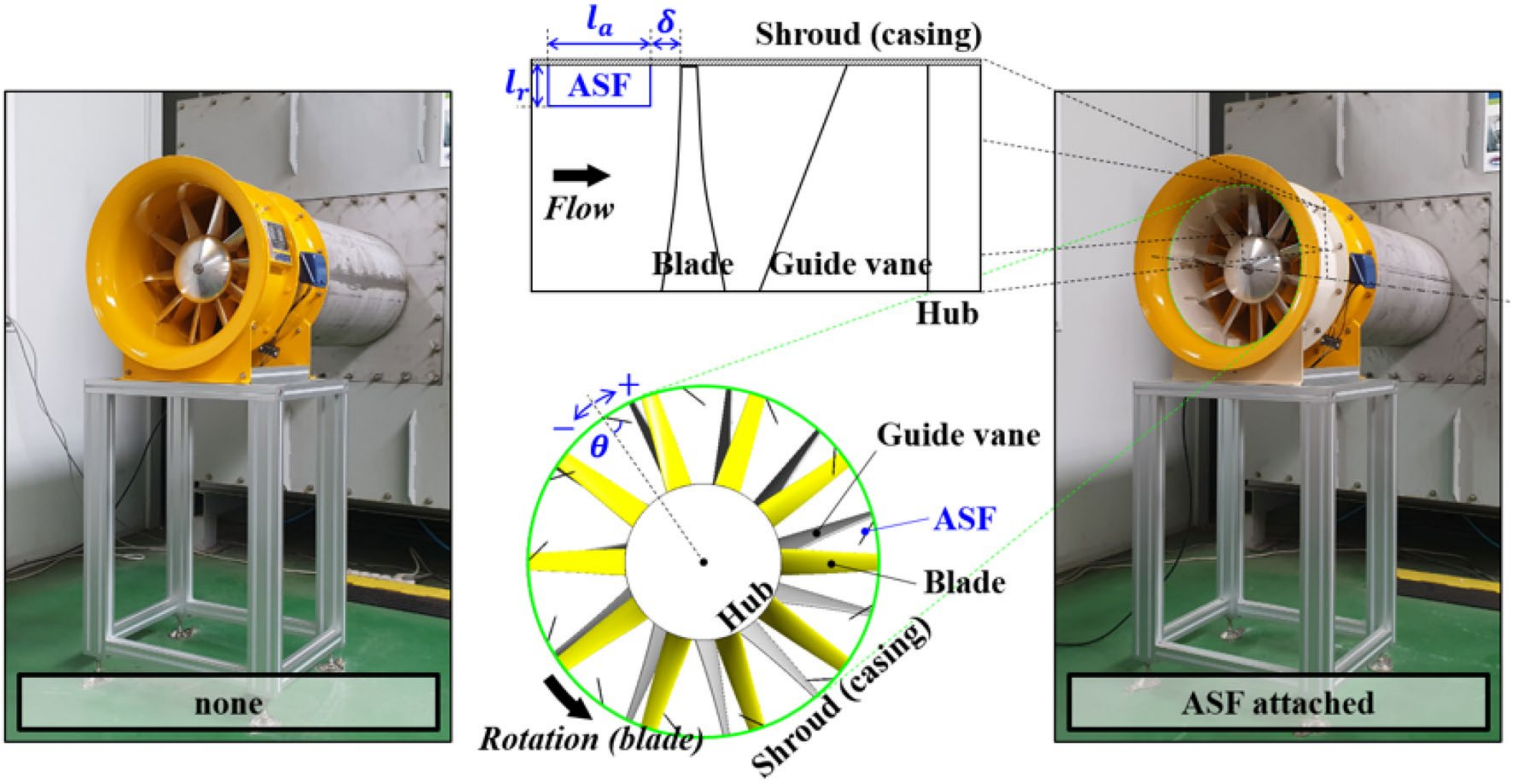
Photograph and schematic drawing for design parameters: case of none (left); case of ASF attached (right); meridional plane for radial length (${l}_{r}$), axial length (${l}_{a}$), and axial gap ($\delta$) (mid-top); front view for number of fins ($Z$) and tangential angle ($\theta$) (mid-bottom).

**Figure 3:**
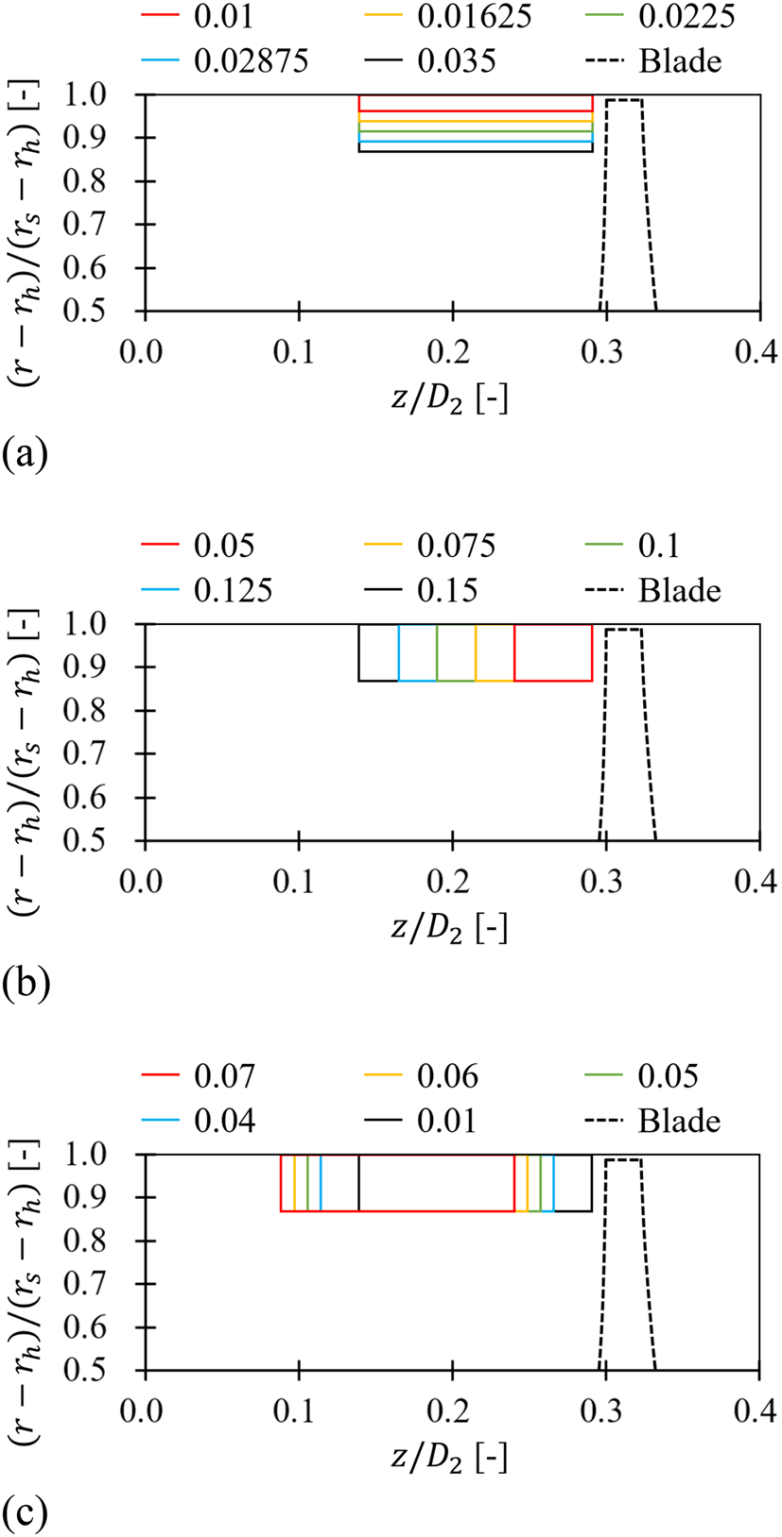
Configuration for (**a**) radial length (${l}_{r}$), (**b**) axial length (${l}_{a}$), and (**c**) axial gap ($\delta$).

Figure 2 shows a photograph of each axial fan connected to the test facility: the case of ‘none’ represents a typical assembly as in Fig. 1; the top and bottom of the schematic drawing in the middle indicate the design parameters of ASF on the meridional and front view, which were enlarged from the case of ‘ASF attached’. Three parameters could be demonstrated from the meridional plane: radial length (${l}_{r}$); axial length (${l}_{a}$); axial gap ($\delta$). From an empirical perspective, $\delta$ would be the most critical parameter; it was based on the fact that the backflow from the blade LE mainly causes instability at low flow rates. In addition, since the backflow occupies a wider region inside the flow passage as the flow rate decreases, ${l}_{r}$ and ${l}_{a}$ were considered as the notable parameters. The number of fins ($Z$) and tangential angle ($\theta$) could additionally be indicated in the front view; here, $\theta$ was artificially assigned, while the right photograph shows the ASF corresponding to $\theta =$ 0°. Finally, the above five parameters were selected as the variables. Table 2 lists the variable range for each parameter: one-factor analysis was performed for each variable based on the dimensions marked as a reference set (^*^); the configuration corresponding to the reference set is shown in the right photograph (Fig. 2); ${D}_{2}$ denotes the fan (outlet) diameter. ${l}_{r}$, ${l}_{a}$, and $\delta$ that can be depicted on the meridional plane are presented for each variable range in Fig. 3. $Z$ was analyzed while maintaining circumferentially arranged symmetry with equal pitch, and $\theta$ was examined while maintaining the two-dimensional geometry without curvature. Meanwhile, the ellipse ratio was selected as 1 (semi-circle) for both LE and TE. The thickness was designed to be constant in terms of general application; it was the same dimension as that of the guide vane in this study.

**Table 2:** Variable range of each design parameter for the anti-stall fin (ASF).

Parameter [unit]	Value					Section
Radial length (${l}_{r}/{D}_{2}$) [−]	0.01	0.01625	0.0225	0.02875	0.035*	“Radial length” section
Axial length (${l}_{a}/{D}_{2}$) [−]	0.05	0.075	0.1	0.125	0.15*	“Axial length” section
Axial gap ($\delta /{D}_{2}$) [−]	0.01*	0.04	0.05	0.06	0.07	“Axial gap” section

Parameter [unit]	Value					Section
Number of fins ($Z$)	4	7		10	13*	“Number of fins” section
Tangential angle ($\theta$) [°]	$\pm$ 0*	$\pm$ 30		$\pm$ 45	$\pm$ 60	“Tangential angle” section

*Reference set.

## Computational setup

### Governing equation and turbulence model

The Reynolds-averaged Navier–Stokes (RANS) equations were solved in the three-dimensional flow field and were discretized as the finite volume method; the conservation of energy was ignored because this study stands for the iso-thermal (25 °C) condition. The conservation of mass (1) and momentum (2) could be:

1 $$\frac{\partial \rho }{\partial t}+\frac{\partial (\rho {U}_{i})}{\partial {x}_{i}}=0$$

2 $$\frac{\partial (\rho {U}_{i})}{\partial t}=-\frac{\partial P}{\partial {x}_{i}}+\frac{\partial }{\partial {x}_{j}}\left[\mu \left(\frac{\partial {U}_{i}}{\partial {x}_{j}}+\frac{\partial {U}_{j}}{\partial {x}_{i}}-\frac{2}{3}\frac{\partial {U}_{r}}{\partial {x}_{r}}{\delta }_{ij}\right)\right]+\rho {F}_{i}$$

where $t$, $U$ (could be substituted as $V$ or $W$), $x$ (could be substituted as $y$ or $z$), and ${F}_{i}$ denote the time, velocity, coordinate, and body force, respectively, and the terms in square brackets denote the viscous stress tensor (${\tau }_{ij}$); these are substituted only for the governing equation. Since the maximum Mach number at the blade tip was estimated as 0.09 at 25 °C (subsonic flow; Mach number $<$ 0.3), there was no change in density over time. Meanwhile, a high-resolution discretization method was adopted based on the second-order approximation, and the root means square (RMS) residuals of mass and momentum were kept within $1.0\times {10}^{-4}$ and $1.0\times {10}^{-5}$.

In terms of turbulence model, the $k$–$\omega$-based shear stress transport standard (SST Std.) model is known to be suitable for rotating machinery: it had been developed to provide accurate predictions in adverse pressure gradients, especially for onset and amount of the flow separation; however, flow separation from smooth surfaces could be exaggerated under the influence of adverse pressure gradients because it included transport effects to the eddy-viscosity formulation^13^; the predicted flow separation is usually overestimated. To enhance turbulence levels in the separating shear layers emanating from walls, a modified SST model was suggested so-called ‘shear stress transport reattachment modification (SST R.M.)’^14^: this model considered an additional source term for $k$-equation^15,16^ to secure the ratio of turbulence production, which might be greatly exceeded in large flow separation so that it is more suitable to focus on the low flow rates; for turbulence production in $k$-equation, the basic term (${P}_{k}$) and the modified term (${P}_{R.M.}$) could be stated as:

3 $${P}_{k}=\left({\mu }_{T}{S}^{2}-\frac{2}{3}\rho k\frac{\partial {U}_{i}}{\partial {x}_{j}}{\delta }_{ij}\right)$$

4 $${P}_{R.M.}={P}_{k}\mathrm{min}\left[4\mathrm{max}\left(0,\frac{\mathrm{min}({S}^{2},{\Omega }^{2})}{0.09{\omega }^{2}}-1.6\right),1.5\right]\mathrm{tanh}\left[{\left(\frac{k}{10\omega \nu }\right)}^{2}\right]$$

where ${\mu }_{T}$, $S$, $k$, $\Omega$, $\omega$, and $\nu$ denote turbulent viscosity, magnitude of strain rate ($\sqrt{2{S}_{ij}{S}_{ij}}$), turbulence kinetic energy, magnitude of vorticity rate ($\sqrt{2{\omega }_{ij}{\omega }_{ij}}$), turbulence eddy frequency, and kinematic viscosity coefficient, respectively. Empirically, the SST Std. and R.M. models had little difference on weak separation such near the design flow rate^17^; the source term had a conditional effect on flow separation. It was also introduced that the effect is remarkable when the grid system is coarse, but it seemed that the effect can be notable even when the grid system is quite fine as in this study; in the validation step for this study, the SST Std. model obtained significantly different gradients near the stalling flow rates containing the positive gradients on the $Q$–$P$ curve, whereas the SST R.M. model derived a relatively accurate prediction^18^. Although the SST R.M. model should not be understood as upward compatibility to the SST Std. model, finally, the SST R.M. model was applied in this study. Meanwhile, the turbulence intensity (${T}_{u}$) and the Reynolds number (Re) were approximately 4.84% on the inlet boundary and 247,763 for the ideal ${c}_{m}$, at each design flow rate for the case of none.

### Boundary condition and grid system

**Figure 4:**
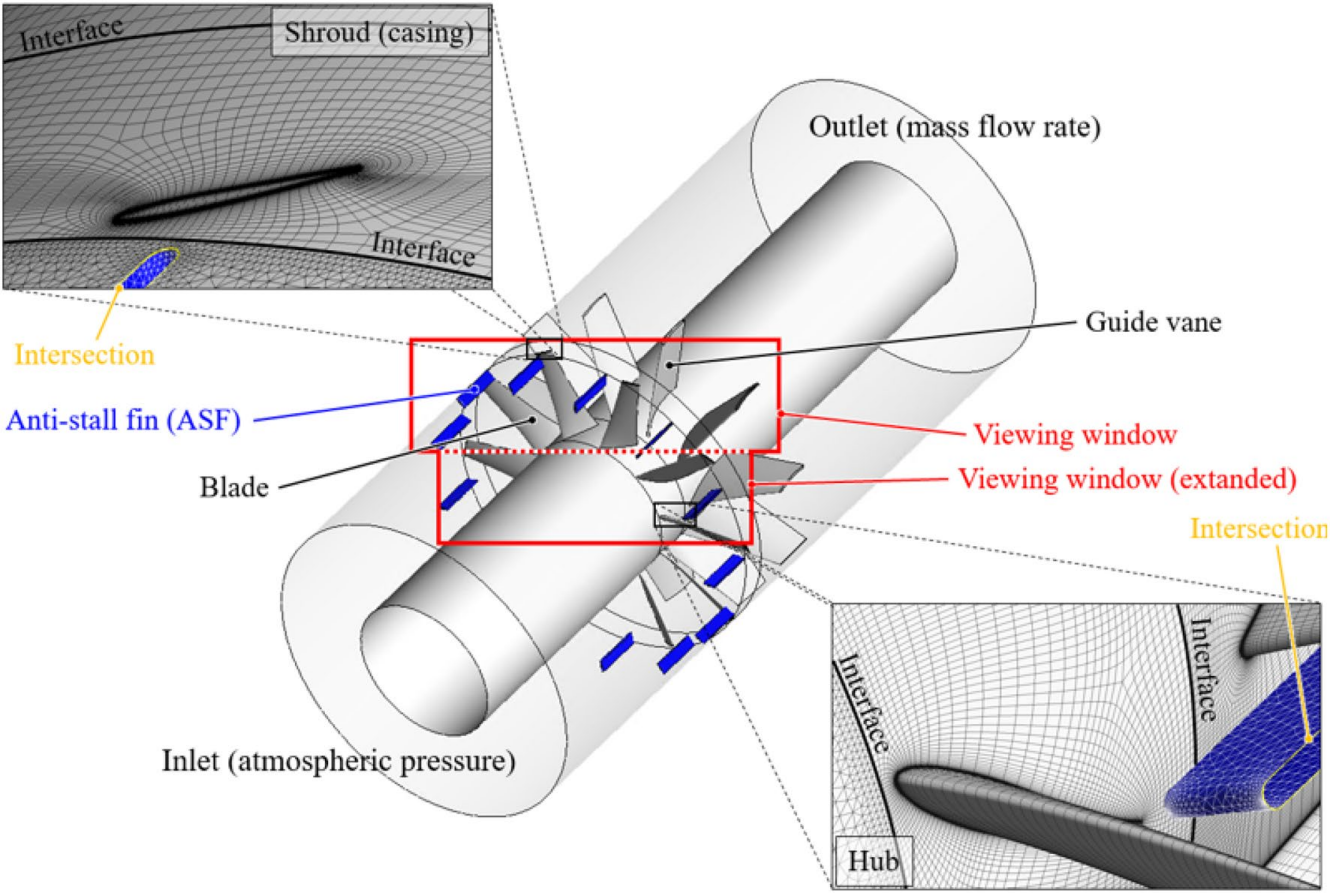
Computational domain and grid system with viewing window for post process.

The whole flow passage is shown in Fig. 4: the inlet passage included the ASFs and was extended to account for unfavorable flow patterns under the stalling flow rates; the rotating passage included the blades, and the counter-rotating condition was given to the shroud wall; the outlet passage included the guide vanes; the stage (mixing-plane) method was applied to each interface. Here, the bell mouse and hub cap could not be considered because their effects were insignificant compared to the straightly extended passage^19^; the total pressure difference between the inlet and outlet on the straightly extended passage did not show a notable deviation from the total pressure derived from the experimental test, which would be further verified with the performance curve in Fig. 7. The wall function was selected as automatic, and the boundary walls were treated as smooth and non-slip conditions.

**Figure 5:**
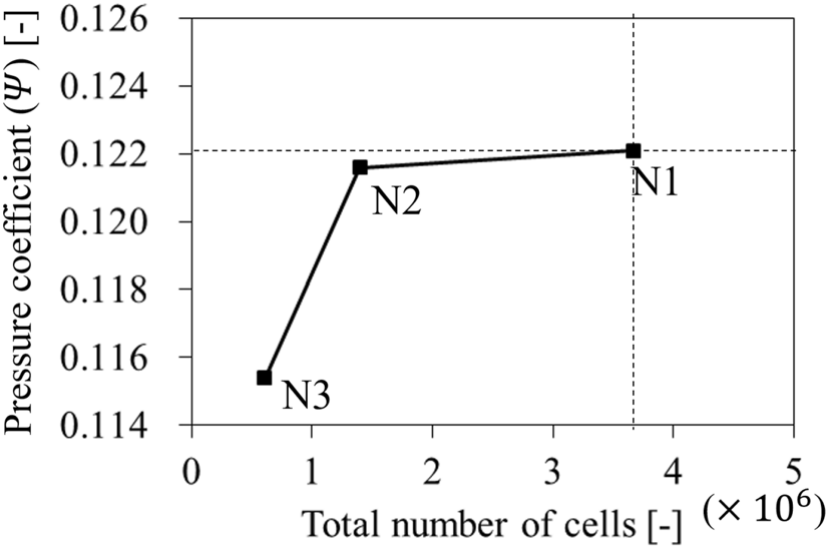
Result of grid test based on grid convergence index method.

The inlet passage was composed of a tetrahedral type, and the rotating and outlet passages were filled with a hexahedral type (see the enlarged window in Fig. 4). The grid test was conducted as shown in Fig. 5; it was at the design flow rate for the case of none. A grid refinement technique (grid convergence index; GCI), which was established by Roache^20^, was employed to quantify the grid convergence. As a result, the convergence corresponding to the N1 set was evaluated as a value, 0.000297, which was considerably lower than the self-proposed criteria^21^; the numerical results were hardly affected with the N1 set, and the grid system was applied with the same topology corresponding to N1 set.

### Other information

A commercial software, ANSYS CFX 19.1, was employed for simulations. The workstation has the specifications as follows: Intel® Xeon® CPU E5-2680 v2; clocked at 2.80 GHz with dual processor; random access memory with 80 GB; 64-bit operating system; parallel computations. The computational time for a set of steady-state simulations was approximately 26 h.

## Results

### Effect of anti-stall fin

#### Experimental validation

**Figure 6:**
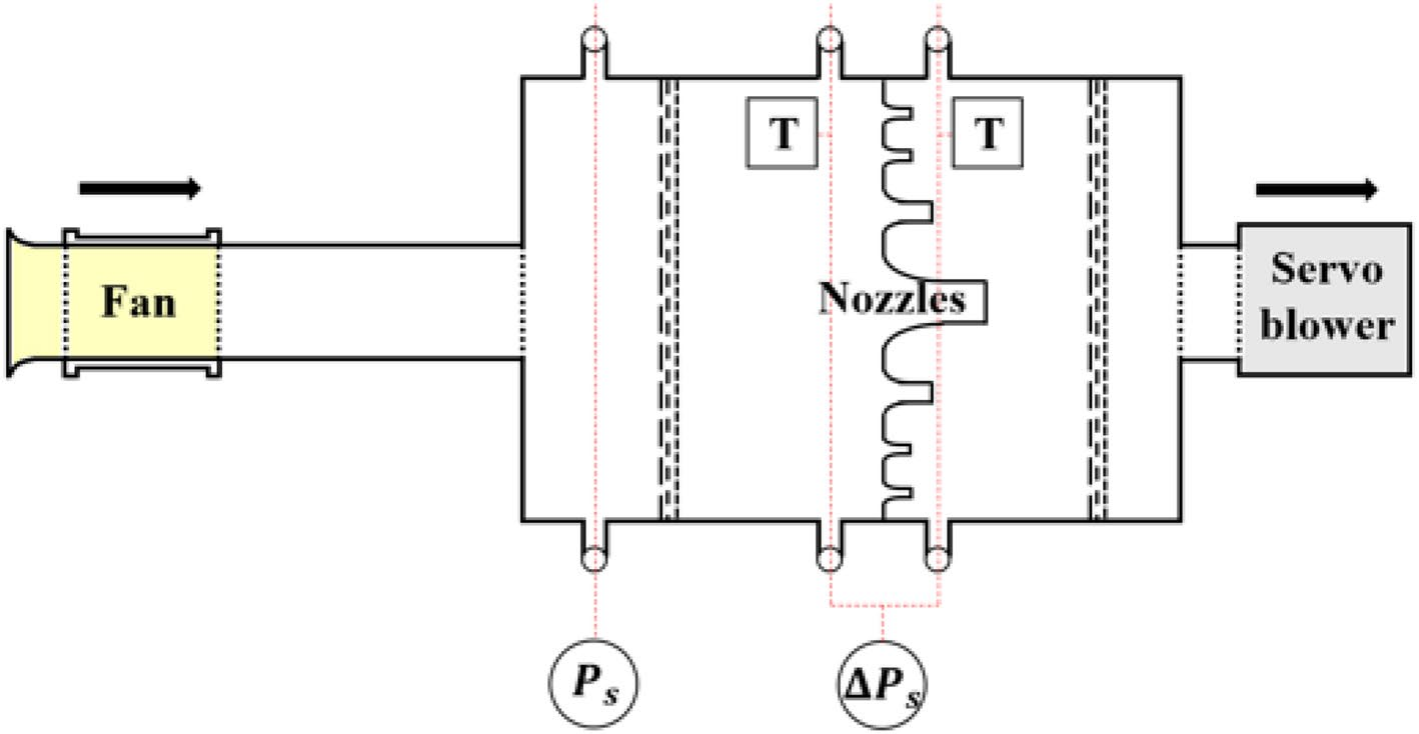
Schematic diagram of experimental test facility for outlet chamber setup.

The experimental process and facility of this study fully complied with the international standard^22^. As shown in Fig. 6 (or Fig. 2), the outlet chamber setup was adopted, and a straight duct that has twice the axial length for the fan diameter was connected between the fan outlet and chamber inlet. The flow settling means inside the chamber had secured the proper porosity^23^. Relative humidity, barometric pressure, and dry-bulb temperature were measured to calculate the density. The density and rotational speed were respectively converted as the same values ​​with the computational setup. The flow rate was adjusted with nozzles and was calculated from the differential pressure ($\Delta {P}_{s}$); the flow rate range that could not be measured with a combination of nozzles was handled with a servo blower behind the chamber, and this was necessary to compensate for the pressure loss contained by the nozzles in the system. The pressure and rotational speed were measured with pressure manometers and a laser tachometer (or a stroboscope). The uncertainty for pressure manometers, stroboscope, and dry-bulb temperature detectors was 0.001–0.005 kilo-pascals for the range of 0–1.33 kilo-pascals, 0.1–1 revolutions-per-minute for the range of 40–35,000 revolutions-per-minute, and 0.07 °C for the range of 0–60 °C, respectively.

**Figure 7:**
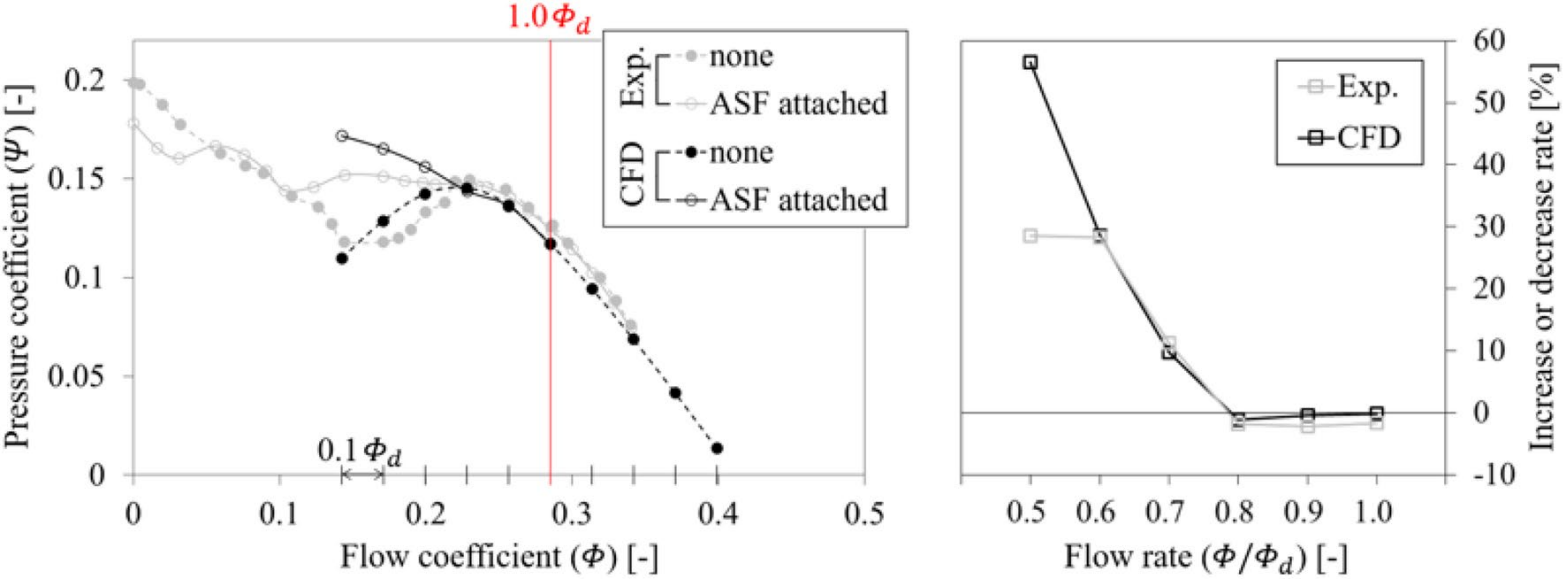
$Q$–$P$ curve for each case of none and ASF attached (left) and increase or decrease rate of total pressure rise with ASF (right).

Figure 7 represents the $Q$–$P$ curve for each case of none and ASF attached (left), and the increase or decrease rate of total pressure rise for the case of ASF attached (right); here, ASF corresponding to the reference set in Table 2 was compared preferentially. In the case of none, the positive gradients were included in the stalling flow rates less than 0.8 ${\varPhi }_{d}$. However, in the case of ASF attached, the positive gradients contained in the case of none were completely reversed to become negative. The ASF-attached axial fan stably recovered performance degradation in the stalling flow rates and allowed to form negative gradients to 0.5 ${\varPhi }_{d}$. Although it would be a strict declaration, the ASF's functional limitation should be evaluated by determining whether the $Q$–$P$ curve forms negative gradients in the flow rate range over 0.5 ${\varPhi }_{d}$.

#### Anti-stalling mechanism

**Figure 8:**
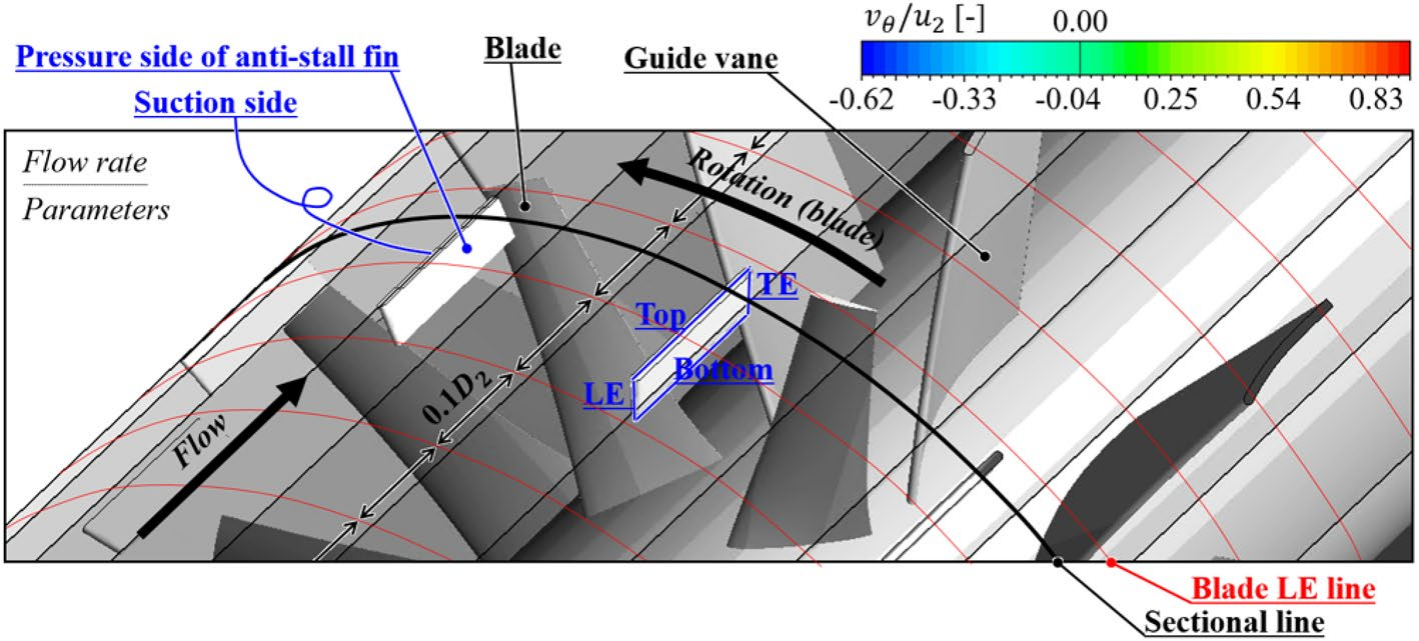
Guide of viewing window in Fig. 4.

**Figure 9:**
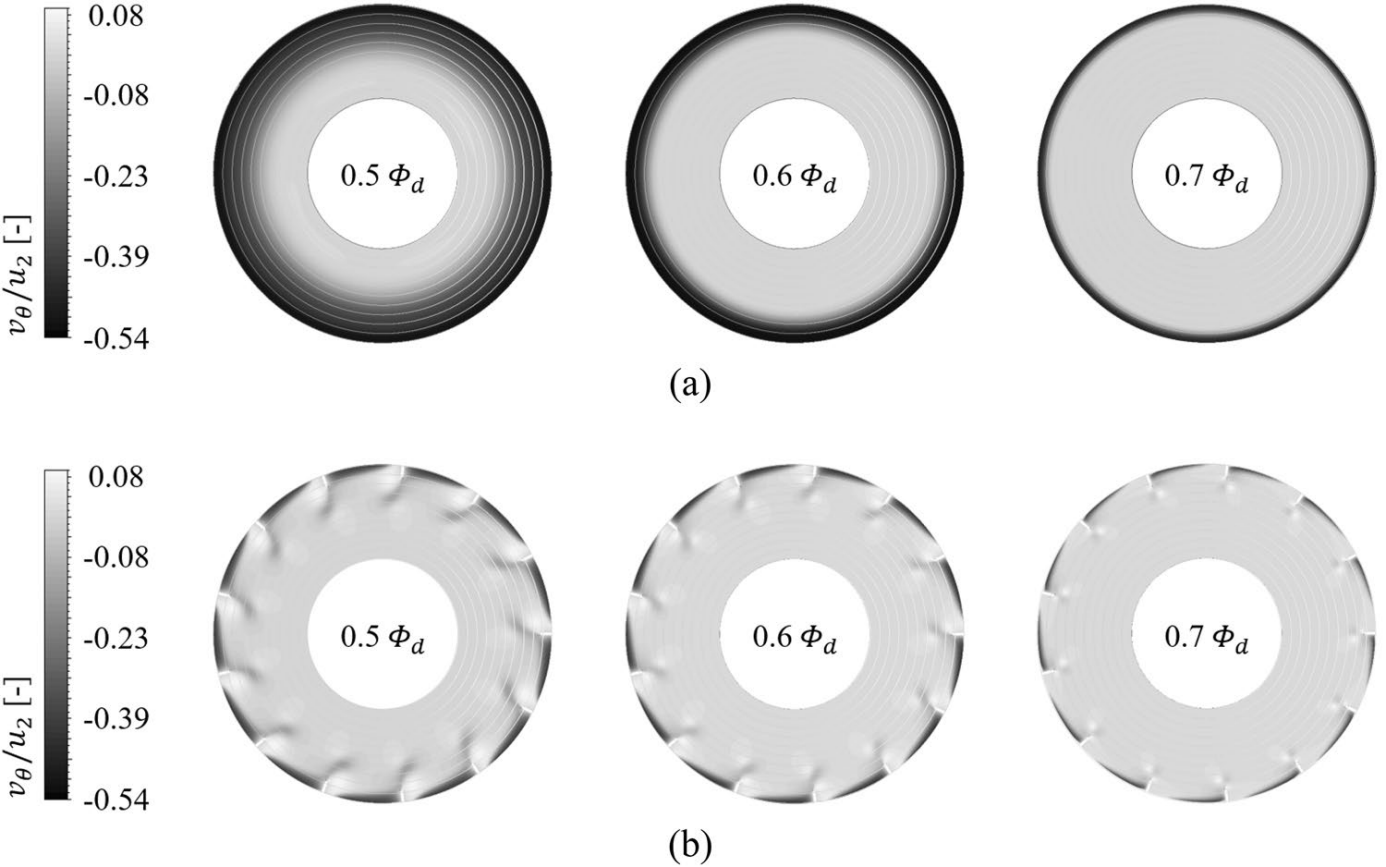
Circumferential velocity (${v}_{\theta }$) contour on sectional plane for each (**a**) case of none and (**b**) ASF attached (0.5–0.7 ${\varPhi }_{d}$).

**Figure 10:**
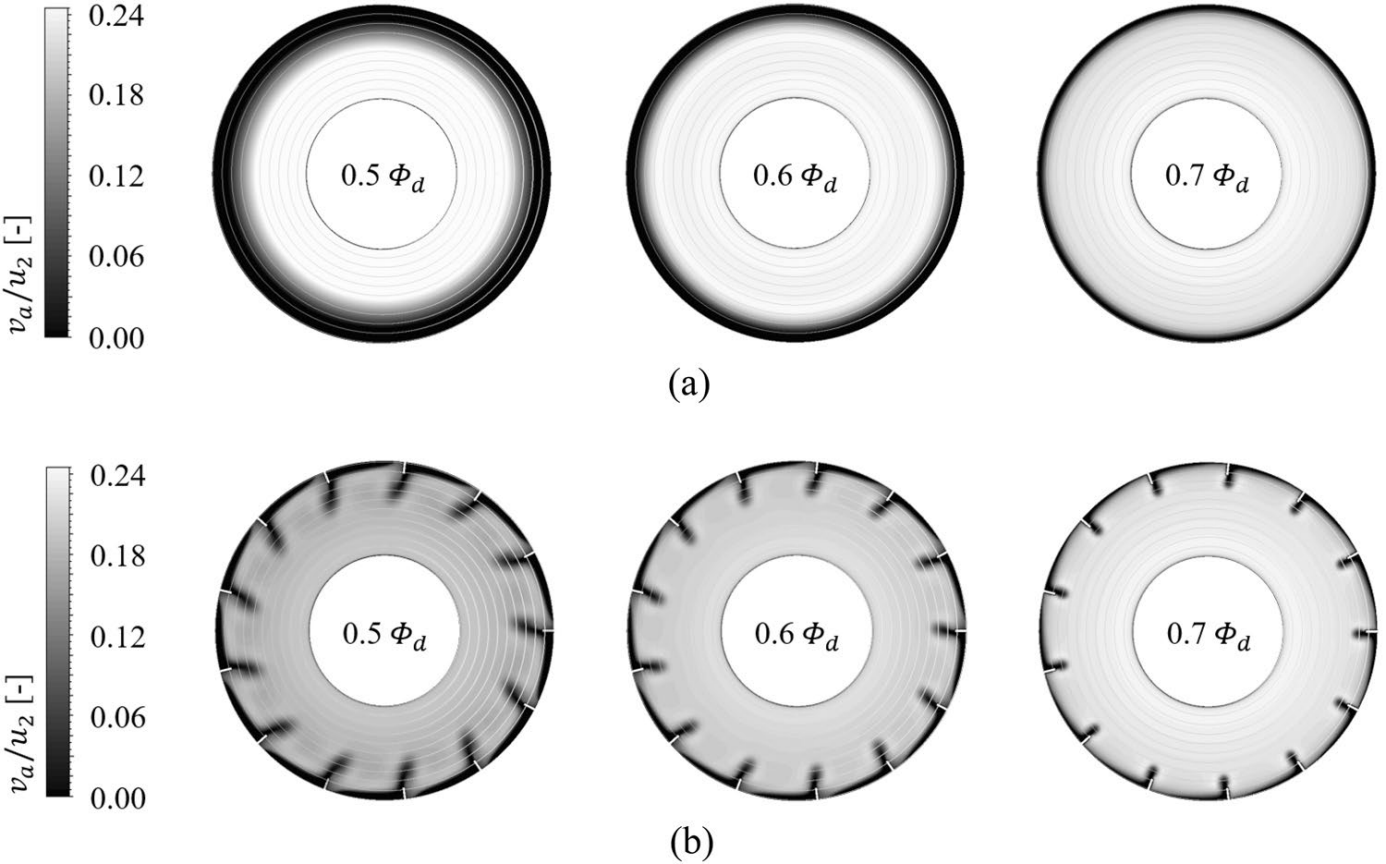
Axial velocity (${v}_{a}$) contour on sectional plane for each (**a**) case of none and (**b**) ASF attached (0.5–0.7 ${\varPhi }_{d}$).

**Figure 11:**
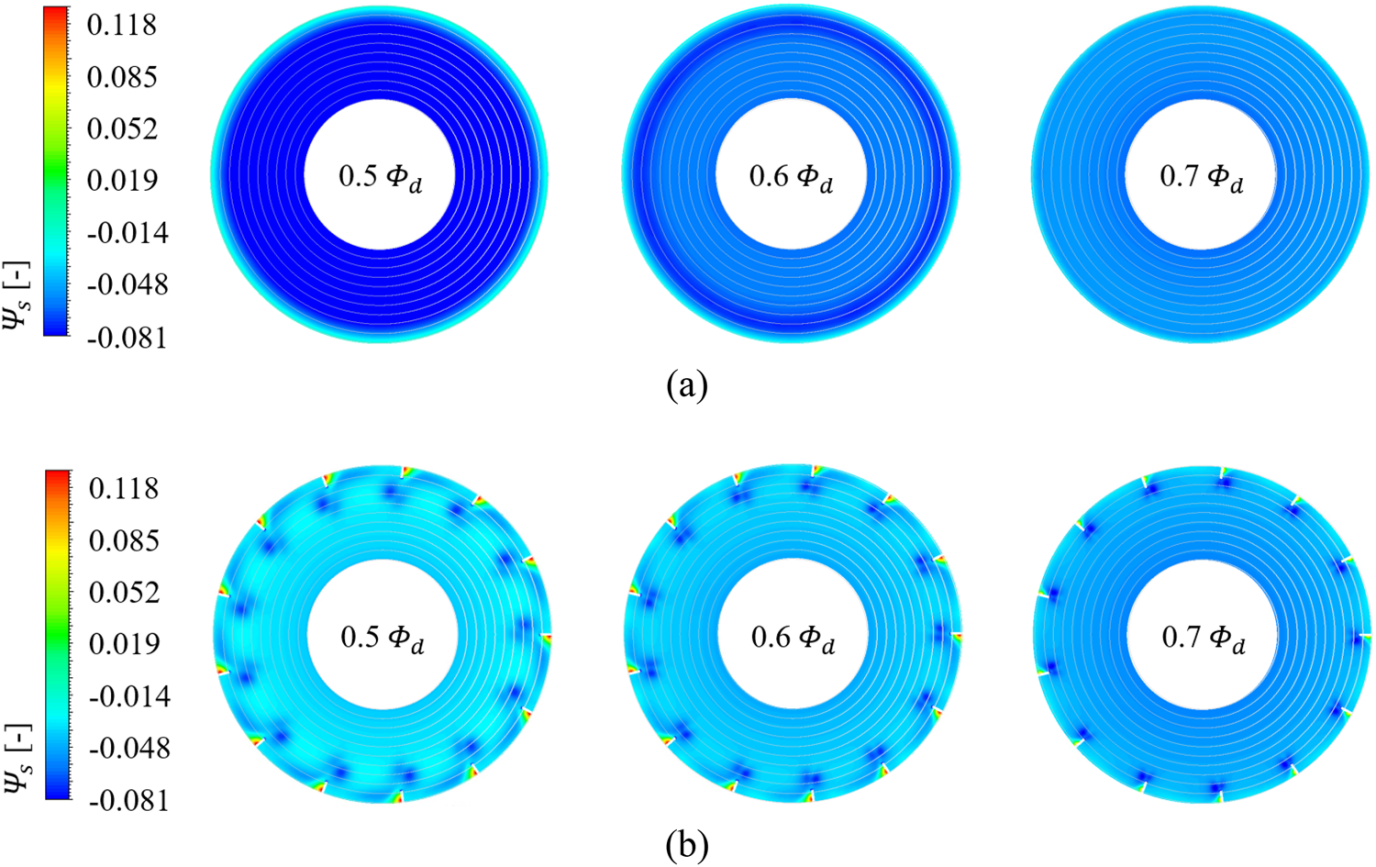
Static pressure (${\varPsi }_{s}$) contour on sectional plane for each (a) case of none and (b) ASF attached (0.5–0.7 ${\varPhi }_{d}$).

Each contour for circumferential velocity (${v}_{\theta }$), axial velocity (${v}_{a}$), and static pressure (${\varPsi }_{s}$) on the sectional plane near the blade LE is shown in Figs. 9, 10, and 11, as the front view: the sectional plane is based on the sectional line in Fig. 8; Fig. 8 is an enlarged view for the viewing window in Fig. 4; Figs. 9, 10, and 11 have each legend on the left, and the legend on Fig. 8 is for subsequent discussion regarding $Q$-criterion; the circumferential velocity contour (Fig. 9) indicates a higher value as it becomes stronger against the blade's rotational direction, and the axial velocity contour (Fig. 10) indicates a higher value as the component toward the downstream becomes stronger; concentric circles in each sectional plane mark every 0.1 from the hub (0) to the shroud (1). In the case of none, each negative portion of ${v}_{\theta }$ and ${v}_{a}$ (Figs. 9a, 10a) were gradually developed over the thicker span as the flow rate decreased. Here, the portion of negative ${v}_{\theta }$ was thicker than that of negative ${v}_{a}$ at each flow rate point, i.e., ${v}_{\theta }$ contained a backward (backflow) and forward components from the shroud to the deeper spans; this means recirculation in the axial direction was formed with rotation in the circumferential direction. However, these flow patterns were mostly controlled by ASF (see Figs. 9b, 10b). This could be confirmed as the main cause that ASF could suppress the positive gradients in the stalling flow rates. In Fig. 11a for the case of none, the static pressure decreased as the flow rate decreased. From a theoretical perspective, a decrease in flow rate means an increase in inlet static pressure; however, as shown in Fig. 10a, the backflow could act as a blockage in the flow passage, causing an increase in ${v}_{a}$ of main stream. On the other hand, in Fig. 11b for the case of ASF attached, the static pressure increased as the flow rate decreased. Meanwhile, each pressure and suction side of ASF (Fig. 8) could be identified with Fig. 11b.

**Figure 12:**
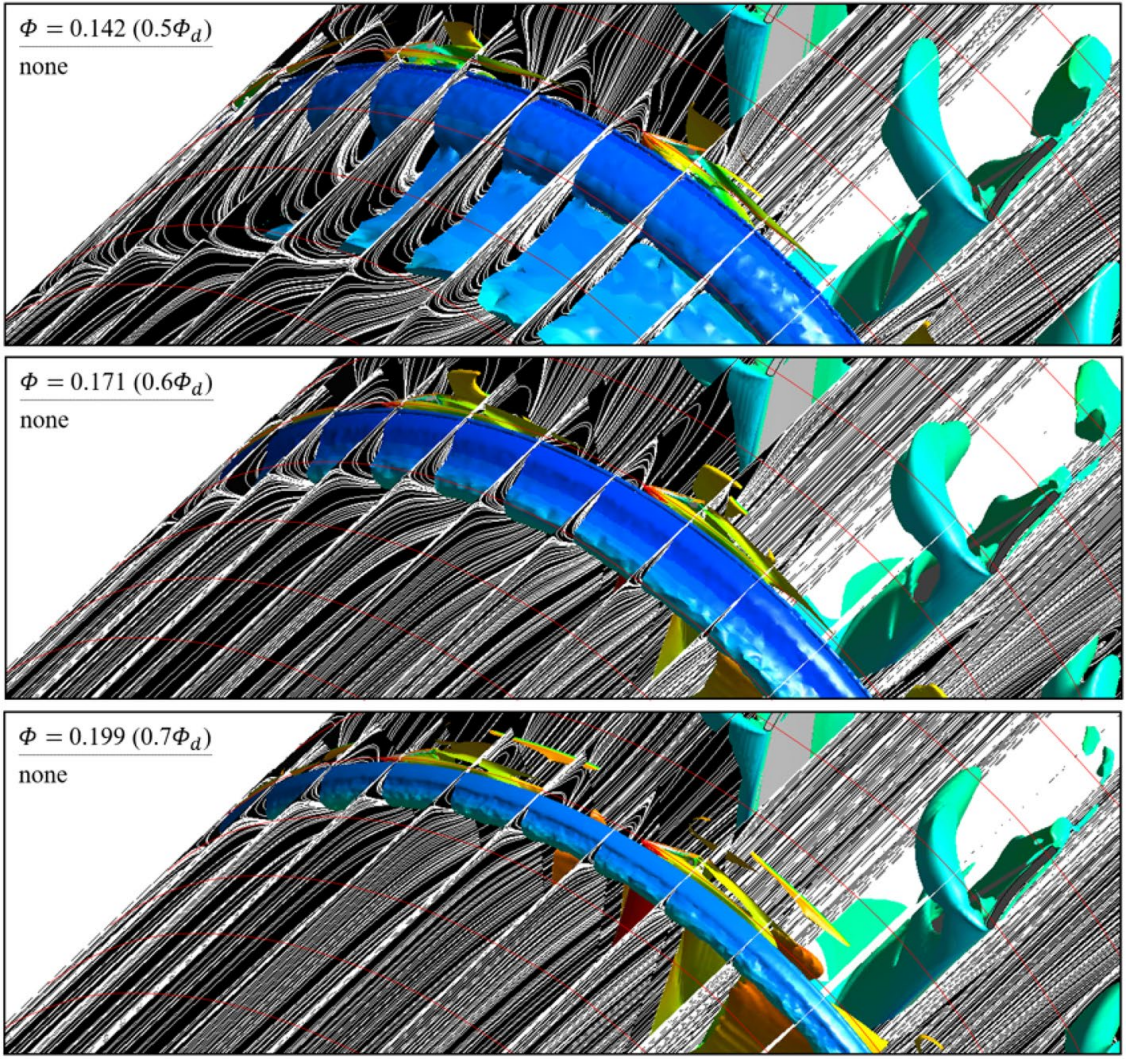
Internal flow field with limiting streamlines and $Q$-criterion for case of none (0.5–0.7 ${\varPhi }_{d}$).

**Figure 13:**
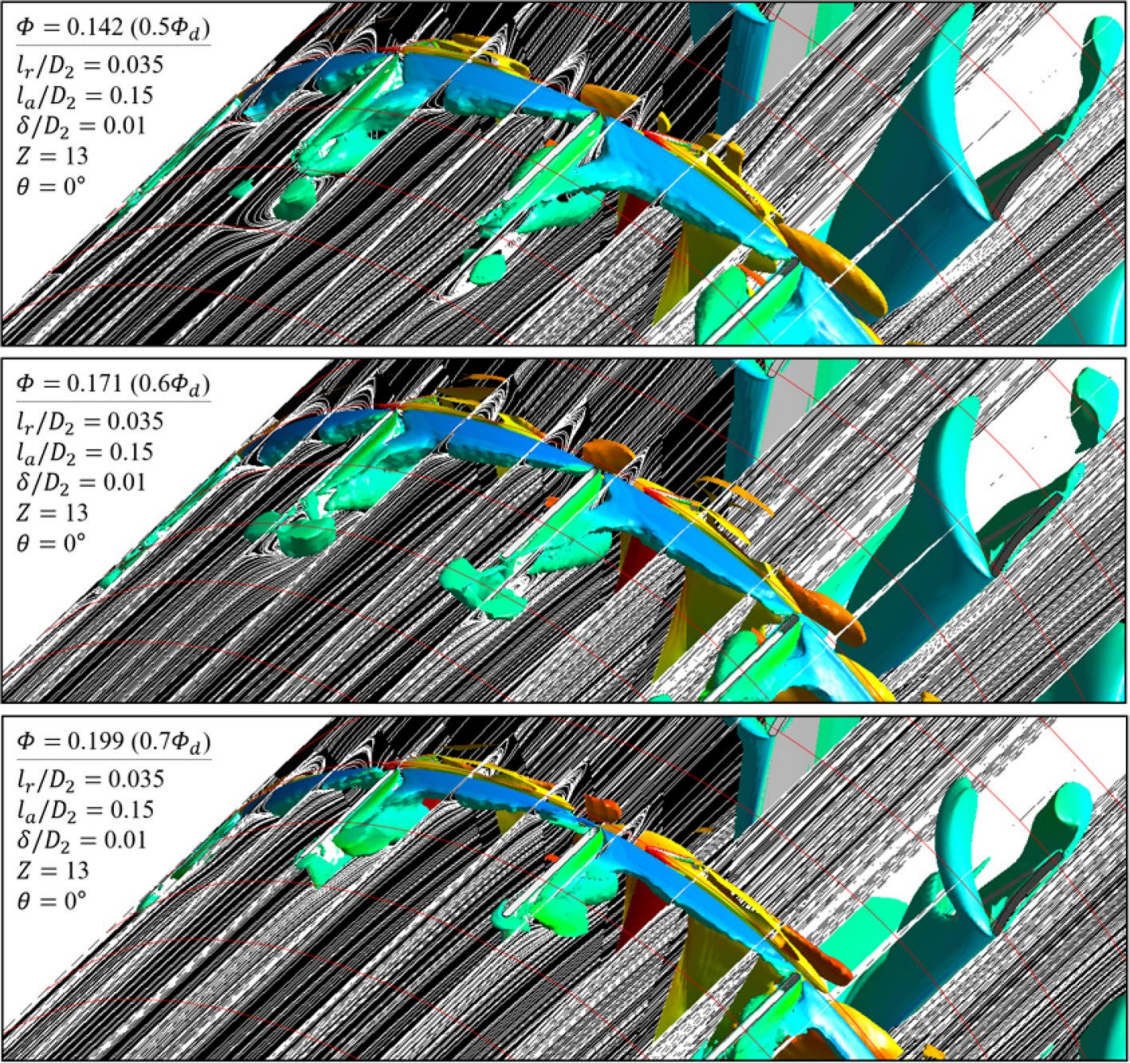
Internal flow field with limiting streamlines and $Q$-criterion for case of ASF attached (0.5–0.7 ${\varPhi }_{d}$); anti-stalling mechanism.

As further details for the internal flow field, Figs. 12 and 13 were illustrated: it was based on the guide in Fig. 8; the red-circumferential lines denote the axial coordinates every 0.1 ${D}_{2}$, and the blade LE line corresponds to 0.3 on the $x$-axis in Fig. 3; the limiting streamlines, which have no legends (white), were plotted on the imaginary planes passing through the axis; the imaginary planes treated as transparent in Fig. 8, but opaque (black) in Figs. 12 and 13; a vortex identification method ($Q$-criterion^24^), was employed with iso-surface that was coated with a circumferential velocity contour with the legend on Fig. 8, and the circumferential velocity contour indicates a higher value as it becomes stronger against the blade's rotational direction; the figures would be focused on the shroud because it is a three-dimensional view with superimposed imaginary planes. In the case of none (Fig. 12), the backflow (limiting streamline) and rotating components ($Q$-criterion) from the blade LE were strongly developed toward the upstream as the flow rate decreased. As expected, the backflow and rotating components were mostly suppressed in the case of ASF attached (Fig. 13). The vortexes isolated on the ASF's pressure side almost lost their velocity and could not pass to the suction side so that it was difficult to cause any instability inside the flow passage. From the combination with Figs. 9 and 10, the residual backflow and rotating components in the fin-to-fin pitch did not penetrate 0.9 spans or less. From the results in this section, the ASF's mechanism could be stated as follow: prevening the development of backflow and re-directing the circumferential velocity components to the axial direction. Meanwhile, the above footnotes for Figs. 12 and 13 would be equally applied to the figures in the section below.

### One-factor analyses

#### Radial length

**Figure 14:**
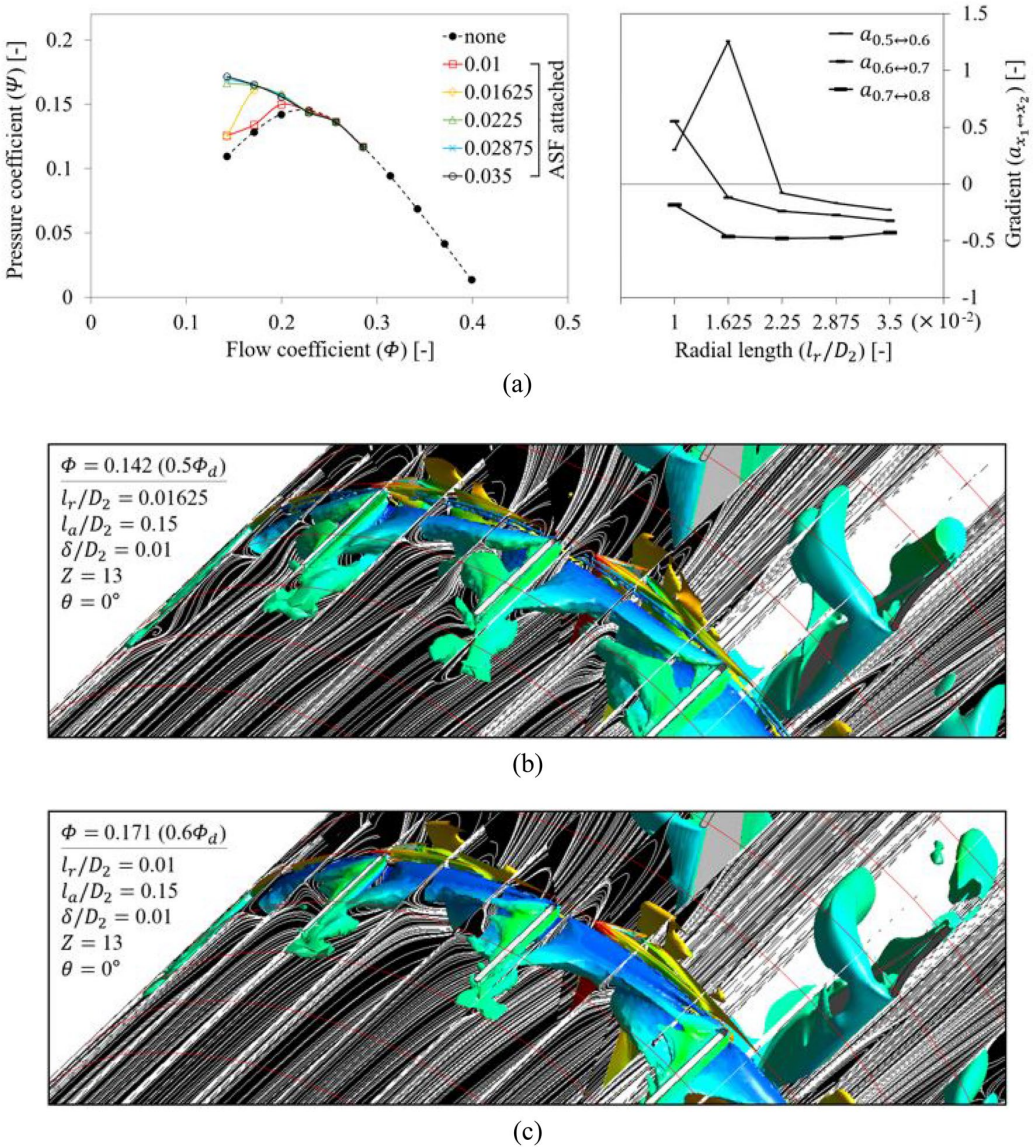
Evaluation for radial length (${l}_{r}$) of ASF: (**a**) $Q$–$P$ curve (left) and the gradient (right) in each flow rate range; (**b**) Internal flow field for ${l}_{r}/{D}_{2}=$ 0.01625 at 0.5 ${\varPhi }_{d}$; (**c**) Internal flow field for ${l}_{r}/{D}_{2}=$ 0.01 at 0.6 ${\varPhi }_{d}$.

From the reference set in Table 2, ${l}_{r}$ was evaluated within the variable range. Figure 14a shows the $Q$-$P$ curve (left) and the gradient (${a}_{{x}_{1}\leftrightarrow {x}_{2}}$; right) in each flow rate range:

5 $${a}_{{x}_{1}\leftrightarrow {x}_{2}}=\frac{{\varPsi }_{x2}-{\varPsi }_{x1}}{{\varPhi }_{x2}-{\varPhi }_{x1}} , \left({x}_{2}={x}_{1}+{10}^{-1}\right)$$

where subscripts ${x}_{1}$ and ${x}_{2}$ denote the multiple of normalized flow rate based on the design flow rate. The ASF with ${l}_{r}/{D}_{2}=$ 0.01625 lost its function at 0.5 ${\varPhi }_{d}$. This is because the rotating flow components, which should be blocked by the ASF, passed over the ASF's bottom and invaded the suction side (see Fig. 14b). Accordingly, separated vortex cores were twisted together in the fin-to-fin pitch, and its circumferential velocity was higher than the case of reference set at 0.5 ${\varPhi }_{d}$ (Fig. 13). The backflow pattern between the blades and guide vanes was rather similar to the case of none at 0.5 ${\varPhi }_{d}$ (Fig. 12); the backflow became stronger toward downstream. From a similar mechanism (see Fig. 14c), the ASF with ${l}_{r}/{D}_{2}=$ 0.01 lost its function in the flow rate range less than 0.6 ${\varPhi }_{d}$. As a reminder of Table 1, ${\delta }_{t}/{D}_{2}=$ 0.0028, where ${\delta }_{t}$ denotes the tip clearance between a blade tip and casing. In the right graph of Fig. 14a, ${l}_{r}$ became progressively sensitive at lower flow rates. On the other hand, a fairly radical and abrupt tendency was confirmed at the point where the ASF lost its function for ${l}_{r}$. This means that if the ASF has an appropriate ${l}_{r}$, its function can be retained almost unchanged. From the results, the functional limitation for ${l}_{r}$ could be proposed as ${l}_{r}/{D}_{2}=$ 0.0225 or more.

#### Axial length

**Figure 15:**
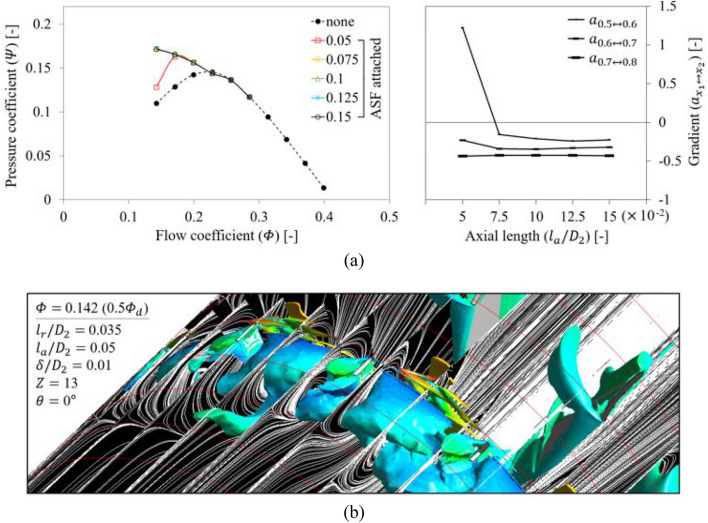
Evaluation for axial length (${l}_{a}$) of ASF: (**a**) $Q$–$P$ curve (left) and the gradient (right) in each flow rate range; (**b**) Internal flow field for ${l}_{a}/{D}_{2}=$ 0.05 at 0.5 ${\varPhi }_{d}$.

The evaluation for ${l}_{a}$ was performed from Fig. 15. In Fig. 15a, the ASF with ${l}_{a}/{D}_{2}=$ 0.05 lost its function at 0.5 ${\varPhi }_{d}$. In particular, the tendency was more dramatic than ${l}_{r}$, i.e., ${l}_{a}$ was difficult to be analyzed as being sensitive to flow rate, and the ASF lost its function more dramatically. In Fig. 15b, the backflow and rotating components were not sufficiently blocked as ${l}_{a}$ was shortened so that the unfavorable flow patterns could pass the ASF's LE. Here, a backflow corresponding to the axial length of approximately 0.2–0.3 ${D}_{2}$ was developed within the fin-to-fin pitch, comparable to the case of none at 0.5 ${\varPhi }_{d}$ (Fig. 12). This was accompanied by the rotating components, as expected. Although somewhat filtered vortexes were confirmed on the ASF's suction side, it was impossible to fulfill the function of ASF. The functional limitation for ${l}_{a}$ could be proposed as ${l}_{a}/{D}_{2}=$ 0.075 or more.

#### Axial gap

**Figure 16:**
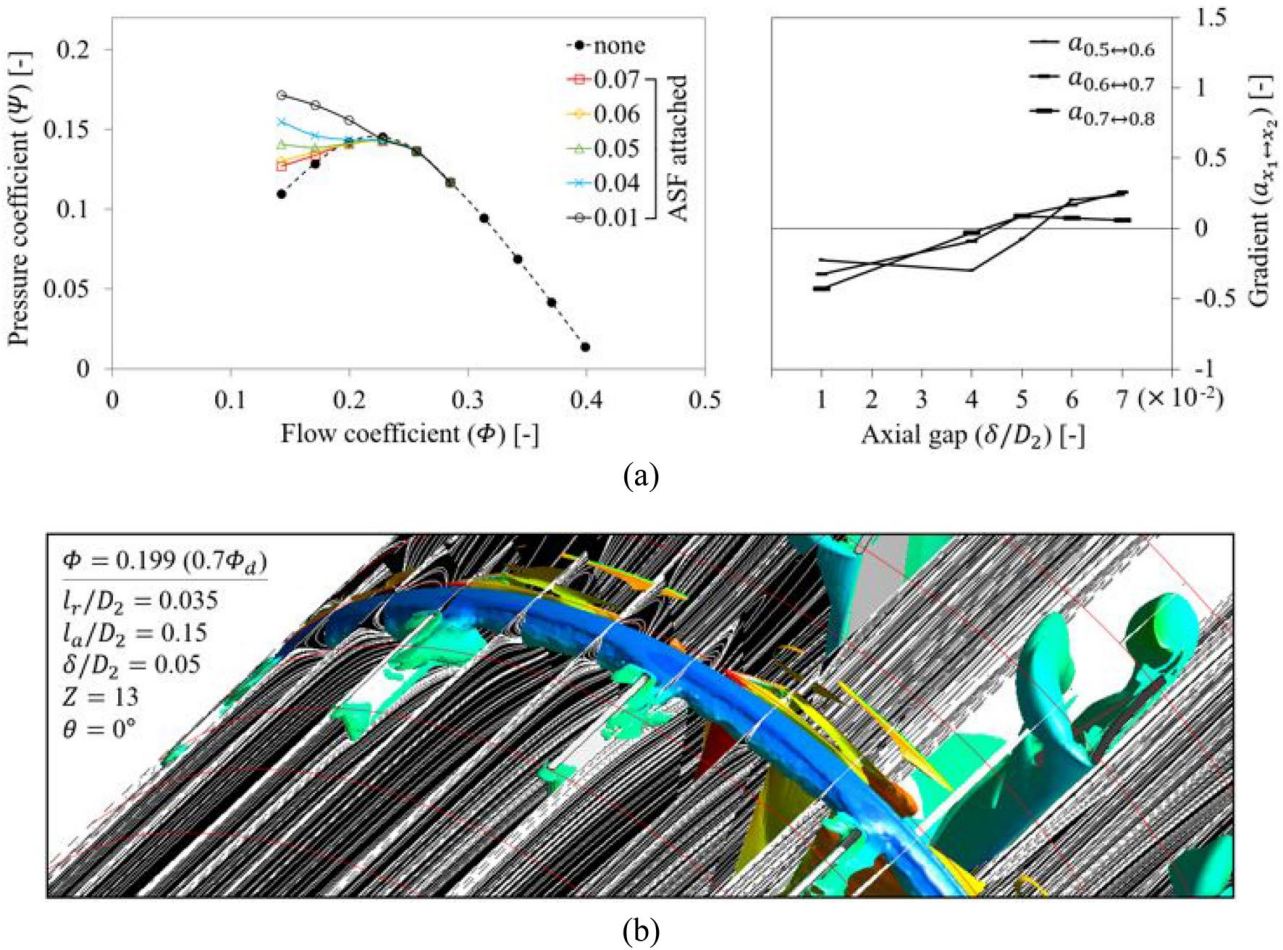
Evaluation for axial gap ($\delta$) of ASF: (**a**) $Q$–$P$ curve (left) and the gradient (right) in each flow rate range; (**b**) internal flow field for $\delta /{D}_{2}=$ 0.05 at 0.7 ${\varPhi }_{d}$.

Figure 16 depicts a one-factor analysis for $\delta$; $\delta$ would be the most critical parameter, as mentioned in Sect. 2. In Fig. 16a, the ASF exhibiting $\delta /{D}_{2}=$ 0.05 lost its function at 0.7 ${\varPhi }_{d}$. The tendency to lose function was gradual, unlike that of ${l}_{r}$ and ${l}_{a}$; $\delta$ may also obtain a radical or dramatic tendency like ${l}_{r}$ and ${l}_{a}$ when it becomes wider beyond the variable range, but it is emphasized that the description was premised on the functional limitation (reversal of gradient) defined in this study. From the empirical background, an increase in $\delta$ suggests that the possibility for suppressing the backflow from blade LE disappears gradually. As $\delta$ increases, the annular vortex core confirmed in Fig. 12 can gradually recover its original intensity for each flow rate. As a result, the formation almost identical to Fig. 12 was confirmed in Fig. 16b, for the same flow rate (0.7 ${\varPhi }_{d}$). The rotating components interfered with the ASF's TE; however, it was insufficient to form a negative gradient on the $Q$–$P$ curve. Even Fig. 16b shows that the whole patterns of the internal flow field looked similar to the case of none; the ASF did not act any performance. Thus, the functional limitation on $\delta$ should be presented as $\delta /{D}_{2}=$ 0.04 or less. As a caution before applying the ASF, a previous analysis on the meridional plane is strongly recommended to prevent contact between the ASF's TE and rotor's LE; the same is true in the case of high-pressure fans (or fluid machinery) that may have problems with thrust.

#### Number of fins

**Figure 17:**
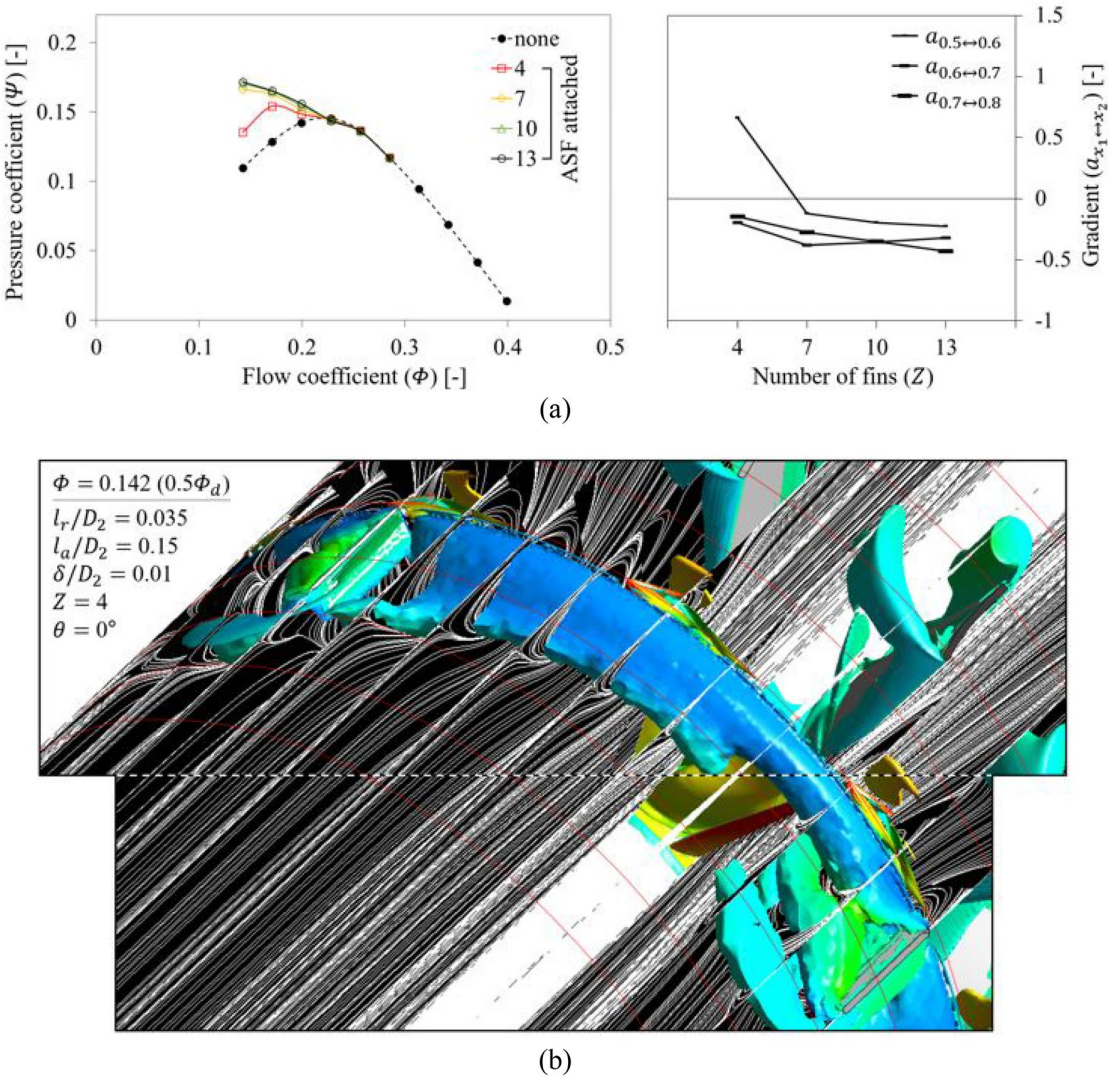
Evaluation for number of fins ($Z$) of ASF: (**a**) $Q$-$P$ curve (left) and the gradient (right) in each flow rate range; (**b**) Internal flow field for $Z=$ 4 at 0.5 ${\varPhi }_{d}$.

From Fig. 17, a one-factor analysis was performed for $Z$. Based on the two graphs in Fig. 17a, the ASF in the case of $Z=$ 4 lost its function at 0.5 ${\varPhi }_{d}$, and the tendency could be analyzed as radical. In Fig. 17b, the viewing window was extended as indicated in Fig. 4 to show one complete fin-to-fin pitch. The annular vortex core within one pitch appeared more restrained than that seen at the same flow rate (0.5 ${\varPhi }_{d}$) in Fig. 12; however, the longer length of one pitch due to the smaller $Z$ caused that the backflow and rotating components could not be sufficiently suppressed. Although the ASF had the same dimensions as the reference set only except for $Z$, strongly developed unfavorable flow patterns were observed in the ASF's pressure side as well as the ASF's suction side. Therefore, the functional limitation on $Z$ was identified as at least 7 or more. For applying the ASF, if an ASF-attached casing duct is separately manufactured to be replaced with the non-ASF casing duct (existing one), i.e., if a method other than on-site welding or fastening is applied, it may be recommended to select $Z$ equal to the number of fan's blades; by positioning the fins into each blade-to-blade pitch, the ASF-attached casing duct can be pushed in a direction parallel to the axis, without disassembling the rotor.

#### Tangential angle

**Figure 18:**
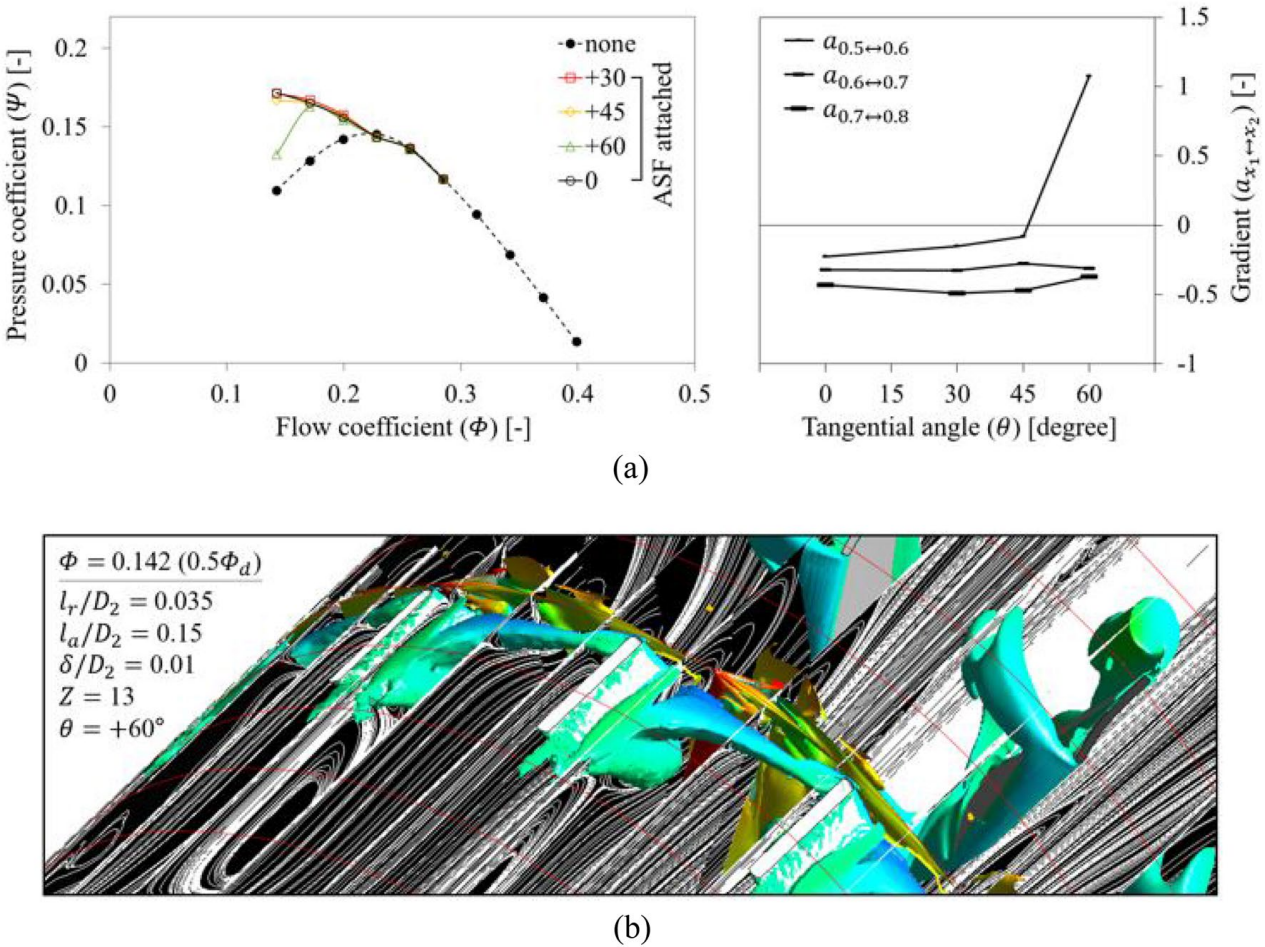
Evaluation for positive-tangential angle ($+\theta$) of ASF: (**a**) $Q$–$P$ curve (left) and the gradient (right) in each flow rate range; (**b**) Internal flow field for $\theta =$ $+$ 60° at 0.5 ${\varPhi }_{d}$.

**Figure 19:**
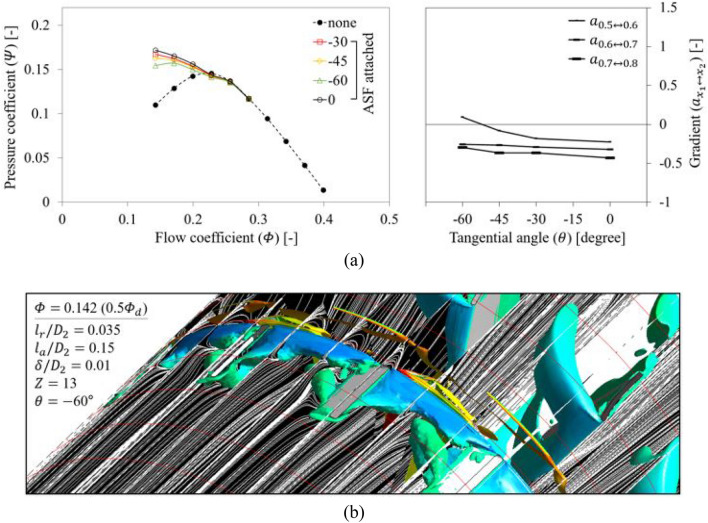
Evaluation for negative-tangential angle ($-\theta$) of ASF: (**a**) $Q$–$P$curve (left) and the gradient (right) in each flow rate range; (**b**) Internal flow field for $\theta =$ $-$ 60° at 0.5 ${\varPhi }_{d}$.

**Figure 20:**
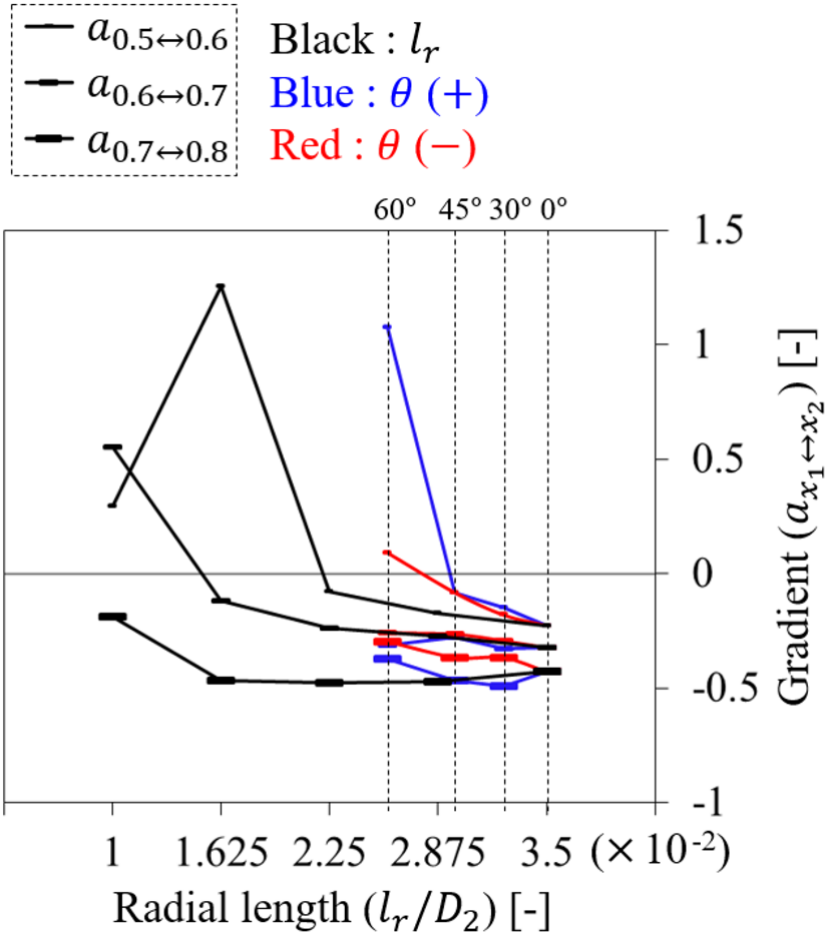
Re-presentation of each right graph in Fig. 14a (black), 18a (blue), and 19a (red) in terms of radial length (${l}_{r}$).

The evaluation for $\theta$ was conducted for each direction (+ and $-$) as indicated in Fig. 2; ‘ + ’ denotes opposition to the blade's rotational direction, and ‘$-$’ denotes the blade's rotational direction. First, as shown in Fig. 18a, the ASF with $\theta =$ + 60° lost its function at 0.5 ${\varPhi }_{d}$ when $\theta$ was given to be opposite to the blade's rotational direction, and the tendency was radical. The assignment of +$\theta$ to the ASF implies that the backflow and rotating components, which should be isolated on the ASF's pressure side, can receive a squeezing effect in the recessed space. Accordingly, although the internal flow pattern in the fin-to-fin pitch (Fig. 18b) closely resembled the condition of the reference set (Fig. 13) at 0.5 ${\varPhi }_{d}$, the backflow and rotating components near the ASF's pressure side caused an additional backflow toward upstream amounting to approximately 0.5 ${D}_{2}$; this backflow was even stronger than that of none at 0.5 ${\varPhi }_{d}$. Figure 19 shows the results in which $\theta$ was given in the same direction as the blade's rotational direction. The ASF with $\theta =$ $-$ 60° lost its function at 0.5 ${\varPhi }_{d}$, whereas the tendency was not radical (see Fig. 19a). In this case, it seemed that the non-isolated flow on the ASF's pressure side extended its force along the circumferential direction rather than upstream and overflowed to the suction side (see Fig. 19b). From the results, the functional limitation for $\theta$ could be presented within $\pm$ 45°. Here, the insensitivity of ASF to $\theta$ can be considered as an additional advantage because it does not require a high concentration during the welding or fastening process. Meanwhile, Fig. 20 depicts the re-presentation of each right graph in Figs. 14a, 18a, and 19a in terms of ${l}_{r}$ in order to consider a correlation between ${l}_{r}$ and $\theta$; the length from the casing to the meanline of ASF's bottom was estimated based on the normal to the casing-fin intersection. The ASF with $\pm \theta$ lost its function at 0.5 ${\varPhi }_{d}$ when ${D}_{2}$-normalized ${l}_{r}$ (${l}_{r}/{D}_{2}$) was estimated as approximately 0.029. Therefore, it is more advantageous to attach the ASF exhibiting correspondingly shorter ${l}_{r}$ without $\theta$ rather than intentional $\theta$.

## Conclusion

The ASF in this study allowed to form negative gradients in the stalling flow rates more than 0.5 ${\varPhi }_{d}$ on the $Q$–$P$ curve. The basic principle was to prevent the development of backflow and re-directed the circumferential velocity components to the axial direction. The functional limitations of ASF were evaluated through each one-factor analysis based on the reference set and were confirmed from the reduction of the volumetric capacity for the back- and rotating-flow on the ASF's pressure side. For ${l}_{r}$, ${l}_{a}$, $Z$, and + $\theta$, the ASF almost retained its function up to the limitation to prevent instability but radically lost its function at a certain flow rate. For $\delta$ and $-\theta$, the ASF gradually lost its function. Each limitation could be summarized as follows:

- $${l}_{r}/{D}_{2}=$$ 0.0225 or more
- $${l}_{a}/{D}_{2}=$$ 0.075 or more
- $\delta /{D}_{2}=$ 0.04 or less (with considerations on the meridional plane and thrust problem)
- $Z=$ 7 or more (with a recommendation of the same number of fan's blades)
- $\theta =$ within $\pm$ 45° (with a recommendation to apply the ASF with correspondingly shorter ${l}_{r}$ rather than assigning $\theta$)

Meanwhile, since the focus of this study was each one-factor analysis based on the reference set, the interactions between parameters could not be addressed; this can be addressed from another focus, such as design of experiments (DOE), sensitivity analysis, and regression equations. In addition, parameters that can be further analyzed may be the ASF's thickness and shape of the edge. The angle that can interfere with the absolute flow angle at the fan blade inlet is not considered because it is against the design concept of ASF.

## Data Availability

The datasets used and/or analyzed during the current study are available from the first or corresponding author on reasonable request.

## List of symbols

${a}_{{x}_{1}\leftrightarrow {x}_{2}}$: Gradient on $Q$–$P$ curve
ASF: Anti-stall fin
$C$: Chord length, $\mathrm{m}$; for the capital $C$ only
${c}_{m}$: Meridional component of absolute velocity, $\mathrm{m}/\mathrm{s}$
CFD: Computational fluid dynamics
$D$: Diameter, m; mainly for shroud (casing)
Exp.: Experimental test
$${F}_{i}$$: Body force, $$\mathrm{kg}\,\mathrm{m}/{\mathrm{s}}^{2}$$
$g$: Acceleration of gravity, $\mathrm{m}/{\mathrm{s}}^{2}$, 9.806
GCI: Grid convergence index
$k$: Turbulence kinetic energy, ${\mathrm{m}}^{2}/{\mathrm{s}}^{2}$
${l}_{a}$: Axial length, $\mathrm{m}$; for ASF
${l}_{r}$: Radial length, $\mathrm{m}$; for ASF
LE: Leading edge
$$N$$: Rotational speed, $$\mathrm{rpm}$$
${N}_{s}$: Specific speed (type number), $\mathrm{dimensionless}$
$P$: Pressure, $\mathrm{Pa}$; mainly for total pressure, but it would denote static pressure with subscript s or specific footnote
PS: Pressure side (surface)
$Q$: Volumetric flow rate, ${\mathrm{m}}^{3}/\mathrm{s}$; it would denote for design flow rate and reference set with subscript d (or des) and ref|Invariant of velocity gradient tensor, $1/{\mathrm{s}}^{2}$
$$r$$: Radius, $$\mathrm{m}$$
${r}^{*}$: Normalized span; hub (0) to shroud (1)
${r}_{h}$: Radius of hub span, $\mathrm{m}$
${r}_{s}$: Radius of shroud span,$\mathrm{m}$
RANS: Reynolds-averaged Navier–Stokes
Re: Reynolds number, $\mathrm{dimensionless}$, $\rho vD/\mu$
R.M.: Reattachment modification
RMS: Root means square
$S$: Blade-to-blade pitch, $\mathrm{m}$|Magnitude of strain rate ($\sqrt{2{S}_{ij}{S}_{ij}}$)
SS: Suction side (surface)
SST: Shear stress transport
$$t$$: Time, $$\mathrm{s}$$
$${T}_{u}$$: Turbulence intensity
TE: Trailing edge
$u$: Circumferential (tangential) velocity, $\mathrm{m}/\mathrm{s}$; mainly for blade tip
$${v}_{a}$$: Axial velocity, $$\mathrm{m}/\mathrm{s}$$
${v}_{\theta }$: Circumferential (tangential) velocity, $\mathrm{m}/\mathrm{s}$
$Z$: Number of fins; for ASF
$$z$$: Axial coordinate, $$\mathrm{m}$$
$\delta$: Axial gap, $\mathrm{m}$; for ASF
$${\delta }_{t}$$: Tip clearance,$$\mathrm{m}$$
$\theta$: Circumferential (tangential) angle, $^\circ$; for ASF
$\nu$: Kinematic viscosity coefficient, ${\mathrm{m}}^{2}/\mathrm{s}$, $\mu /\rho$
$$\rho$$: Density, $$\mathrm{kg}/{\mathrm{m}}^{3}$$, 1.185 at 25 °C
${\tau }_{ij}$: Viscous stress tensor, $\mathrm{N}/{\mathrm{m}}^{2}$
$\varPhi$: Flow coefficient, $\mathrm{dimensionless}$; it would denote for design flow rate and reference set with subscript d (or des) and ref
$\varPsi$: Pressure coefficient, $\mathrm{dimensionless}$; mainly for total head, but it would denote static head coefficient with subscript s or specific footnote; it would denote for design flow rate and reference set with subscript d (or des) and ref
$\Omega$: Magnitude of vorticity rate ($\sqrt{2{\omega }_{ij}{\omega }_{ij}}$)
$\omega$: Angular velocity, ${\text{rad/s}}$, $d\theta {\text{/}}dt$|Turbulence eddy frequency, $\frac{1}{\mathrm{s}}=\mathrm{Hz}$, $k{\text{/[}}\nu \left( {\mu _{T} {\text{/}}\mu } \right){\text{]}}$ where $\mu _{T} = \rho k{\text{/}}\omega$
